# The influence of group membership on the neural correlates involved in empathy

**DOI:** 10.3389/fnhum.2013.00176

**Published:** 2013-05-06

**Authors:** Robert Eres, Pascal Molenberghs

**Affiliations:** School of Psychology, The University of QueenslandSt. Lucia, QLD, Australia

**Keywords:** group membership, social categorization, social neuroscience, empathy, vicarious responses

## Abstract

Empathy involves affective, cognitive, and emotion regulative components. The affective component relies on the sharing of emotional states with others and is discussed here in relation to the human Mirror System. On the other hand, the cognitive component is related to understanding the mental states of others and draws upon literature surrounding Theory of Mind (ToM). The final component, emotion regulation, depends on executive function and is responsible for managing the degree to which explicit empathic responses are made. This mini-review provides information on how each of the three components is individually affected by group membership and how this leads to in-group bias.

In their Perception-Action Model of empathy, Preston and de Waal (2002) state that “the attended perception of the object's state automatically activates the subject's representations of the state, situation, and object, and that activation of these representations automatically primes or generates the associated autonomic and somatic responses, unless inhibited.” Their view of empathy included various phenomena such as emotional contagion, cognitive empathy, guilt, and helping which according to their model all relied on the perception-action mechanism. While typically empathy has been investigated using behavioral paradigms, more recently it is becoming tangible to investigate the neural architecture that underlies this process (Preston and de Waal, 2002; Boston, 2007; Singer and Lamm, 2009; Decety, 2011; Shamay-Tsoory, 2011; Bernhardt and Singer, 2012). Decety (2011) recently proposed a three component basis for empathic experiences, highlighting affective, cognitive, and emotion regulative components. These components are deemed necessary for experiencing empathy where the affective component is identified as a bottom-up, or automatic, process and the cognitive and emotion regulative components are identified as top-down modulators. That is, sharing the pain of others occurs automatically but behavioral responses are differentiated by cognitive factors (for example, perspective taking) and emotion regulative factors (for example, motivation). Social neuroscience has also begun investigating the modulating factors that interfere with empathic responses such as inter-individual differences (Singer et al., 2004; Hein and Singer, 2008), closeness (Beeney et al., 2011), and groups (Ito and Bartholow, 2009; Chiao and Mathur, 2010). Group membership describes a group of people sharing similar and recognizable characteristics where an individual can categorize others as belonging to that particular social group (Abrams, 2012). The focus of the present review is to identify how group membership affects each of the three components of empathy and to illustrate how this accumulates to a biased view of how we see the world.

## Affective empathy: the ability to share the affective states of others

The main problem in understanding empathy from a neuroscience perspective is explaining how we can overcome the physical distance between our brain and that of others. How can we make sure we experience the same emotions as others and how can we understand the emotions of others by just observing their behaviors? Simulation theory suggests that we understand other people's actions and emotions by mirroring their actions and feelings onto our own mind state (Preston and de Waal, 2002; Rizzolatti and Fabbri-Destro, 2008; Keysers and Gazzola, 2009; Rizzolatti and Sinigaglia, 2010). According to the classical view, perception-action coupling of motor actions is supported by mirror neurons located in areas such as the inferior parietal lobule (IPL) and posterior inferior frontal gyrus (Iacoboni et al., 1999; Rizzolatti et al., 2001), however, fMRI studies have shown that additional regions such as superior temporal sulcus (STS), dorsal and ventral premotor cortex and superior parietal lobule are also involved in perception-action coupling of motor actions (Molenberghs et al., 2009, 2010; Caspers et al., 2010).

The human mirror system does not passively respond to the observation of actions but is influenced by the mindset of the observer (Molenberghs et al., 2012c). Crucially for this review, previous studies have shown that group membership can modulate perception-action coupling. For example, a recent fMRI study (Molenberghs et al., 2012b) investigated the effect group membership has on our ability to accurately represent action perception. Participants were randomly divided into red or blue teams and they were told they had to compete against a member of the other team by pressing a button response as quickly as possible. In a subsequent experiment, participants were shown video clips of either in-group or out-group members making button-press responses as quickly as possible in a similar competitive situation, where their job was to identify which team member pressed the button fastest. On average both groups in the video clips pressed the buttons equally fast but behavioral analysis showed that participants responded that their team members pressed the button faster. Additional fMRI analyses showed differential neural activation when presented with actions of in-group members compared with out-group members. That is, for those participants who showed an in-group bias behaviorally (those participants that said their team members were faster), greater activity in the IPL was shown when observing in-group members perform the action compared with members from the out-group (Molenberghs et al., 2012b). The IPL plays an important role in perception action coupling and its modulation by group membership suggests we simulate the actions of in-group members more easily. This is in line with a recent EEG study by Gutsell and Inzlicht (2010), who found larger EEG mu suppression (which has previously been associated with mirror neuron activity) when observing actions of in-group members compared to actions of out-group members. Interestingly, this effect increased with the amount of prejudice toward the out-group (Gutsell and Inzlicht, 2010). This reduced perception-action coupling for out-group members also extends to feelings of empathy. For example in a TMS study, Avenanti and colleagues (2010) found a reduction in motor-evoked potential (MEP) amplitude in the hand of participants (induced by TMS to the contralateral motor cortex) when watching an in-group member being painfully stimulated (compared to touch) but no such effect was found when watching out-group members in pain. This suggests that participants simulated the pain of the in-group member but not the pain of the out-group member.

Though predominantly focused on action-perception, vicarious experiences through mirroring have also been shown to extend to emotion and sensory domains as well (Carr et al., 2003; Keysers et al., 2004, 2010; Keysers and Fadiga, 2008; Keysers and Gazzola, 2009). Observing another person's emotional or sensory state elicits activity in a homologous area in the observer, supporting the notion that we vicariously experience the emotional and sensory states of others and represent these states onto our own emotional and sensory repertoires (Keysers and Gazzola, 2009). Indeed a recent meta-analysis including 125 fMRI studies on the mirror system found that perception-action coupling of emotional expressions through vicarious experience is not limited to the aforementioned mirror areas but also involves brain areas involved in, for example, experiencing pain such as the insula and cingulate cortex (Molenberghs et al., 2009). The role of the mirror system in action understanding and affective empathy is controversial (Saxe, 2005, 2006; Hickok, 2009; Decety, 2010) but our view here is that vicarious responses are at least partially involved in affective empathy through mirroring processes, though we acknowledge that they are only part of the story. For example Decety (2011) views affective empathy more broadly as just mirroring and his model of affective empathy also includes affective arousal which he identifies as “the automatic discrimination of a stimulus as appetitive or aversive, hostile or hospitable, pleasant or unpleasant, threatening or nurturing.”

Neuropsychological evidence suggests that greater vicarious empathic responses are elicited from own-ethnicity members compared with other-ethnicity members (Avenanti et al., 2006, 2010; Ito and Bartholow, 2009; Xu et al., 2009; Chiao and Mathur, 2010; Azevedo et al., 2012; Gutsell and Inzlicht, 2012; Sessa et al., 2013). For example, a recent fMRI study showed that when observing a member of the same ethnicity experiencing painful stimulation, greater activity in the dorsal anterior cingulate cortex (dACC) and anterior insula (AI) were found compared with when a member from a different ethnicity was experiencing pain (Xu et al., 2009). Race, however, is not the only factor to influence empathic responses to in-groups and out-groups. Group membership has also been found to moderate activation of the AI in response to observing painful situations. Hein and Colleagues (2010) showed in their fMRI study that greater activation in the left AI was found when in-group members (those from the same sporting team) received pain compared with out-group members (those from another sporting team). This activity was also found to correlate positively with the willingness to share the pain with an in-group member compared with an out-group member. When and out-group member received pain, rather than an increase in AI activity, more activity occurred in the right ventral striatum [an area typically associated with pleasure and schadenfreude (Singer et al., 2006; Takahashi et al., 2009)], and this activity was negatively correlated with the willingness to share the pain of the out-group member (Hein et al., 2010). In a similar fMRI study, Cikara and colleagues (2011) monitored neural activity when participants watched video clips of two sporting teams (participant favorite vs. other) compete against each other. They found that when the participants' team won, increased activity in the ventral striatum was observed. More importantly, though, when the participants' team lost, greater activity in the AI and dACC were shown suggesting that participants were empathizing with the pain that the players of their favored team felt. However, sharing the emotions with others alone cannot explain the rich experience of empathy. Empathy also involves a cognitive and emotional regulative component.

## Cognitive empathy or the ability to reason about others' mental states

Vicariously sharing other people's emotions helps us partially understand how other people are feeling, but to completely understand the beliefs, desires and intentions of others, one must also reason about the mental state of others. This cognitive aspect of empathy is typically associated with regions associated with mental state reasoning or so called Theory of Mind (ToM) and often involves regions such as the medial Prefrontal Cortex (mPFC), Temporoparietal Junction (TPJ), and adjacent posterior Superior Temporal Sulcus (pSTS) (Amodio and Frith, 2006; Saxe, 2006; Decety and Lamm, 2007; Frith, 2007; Keysers and Gazzola, 2007; Uddin et al., 2007; Shamay-Tsoory et al., 2009; Van Overwalle and Baetens, 2009; Cheon et al., 2010; Shamay-Tsoory, 2011).

Cognitive empathy can also be modulated by group membership. Adams et al. (2009) used an fMRI modified version of the “Reading the Mind in the Eyes Test” (Baron-Cohen et al., 2001) in which participants are presented with pictures of just the eyes of people and participants then have to judge what the person in the picture is thinking or feeling. Adams et al. (2009) used pictures of Asian and Caucasian people and then let native Japanese and white Americans judge the mental state of those people. They found a behavioral intra-cultural advantage for understanding the mental state of in-group members compared to out-group members and showed that this in-group bias was associated with increased activity in the posterior STS. In line with Adams et al. (2009), research surrounding ToM has consistently shown the importance of the STS in understanding the mental states of others (Fletcher et al., 1995; Allison et al., 2000; Gallagher and Frith, 2003; Amodio and Frith, 2006). Similarly, Cheon et al. (2011) found that Korean participants showed more empathy for in-group members experiencing emotional pain than out-group members and that this was related to increased activity in the TPJ. Similar studies have also illustrated the importance of the mPFC in in-group bias. For example, Mathur and colleagues (2010) found increased activation in the mPFC when watching in-group members experience emotional pain compared to out-group members and this increase predicted greater empathy and altruistic motivation for one's in-group. Another fMRI study found mPFC activation when participants watched pictures of social groups but not for extreme low-status groups (Harris and Fiske, 2006).

The mPFC also has an important role in social categorization, with increased activation in this region previously associated with in-group concepts compared to out-group concepts in both existing (Morrison et al., 2012) and newly created groups (Molenberghs and Morrison, 2012). Volz and colleagues (2009) also found that during an fMRI modified version of the minimal group paradigm (Tajfel et al., 1971) high in-group favoritism was associated with increased activation in the mPFC. Taken together, the aforementioned findings suggest that increased activation in cognitive empathy regions are associated with increased understanding of the mental state of in-group compared to out-group members (Adams et al., 2009; Mathur et al., 2010; Cheon et al., 2011), in-group minus out-group social categorization (Volz et al., 2009; Molenberghs and Morrison, 2012; Morrison et al., 2012) and in-group favoritism (Volz et al., 2009), suggesting further the modulating role of group membership on empathic experiences.

## Emotional self-regulation or the control of explicit emotions

To reiterate, affective empathy is partially supported by simulating the emotional states of others whereas cognitive empathy relies partially on understanding another's mental state through cognitive reasoning. Given this capacity to experience the affective and mental states of others, it seems necessary that an additional network be set to moderate the degree to which we experience these effects or explicitly express these states. Without an emotion regulative network, shared emotional states may inhibit our ability to perform tasks that require emotional distance (e.g., a surgeon operating on a child or a defense lawyer supporting a psychopath) or it may interfere with our ability to hide automatic biases (e.g., a parent being derogative to a teacher of a different racial background). Essentially, there needs to be a neural function that inhibits or facilitates empathic responses more explicitly to allow for appropriate functioning in day-to-day life (Decety, 2011). Areas involved with emotion regulation such as the rostral anterior cingulate cortex (rACC), dorsolateral (dlPFC) and ventromedial (vmPFC) prefrontal cortex have previously been shown to modulate the effects of empathy (Amodio et al., 2006, 2008; Cheng et al., 2007; Beer et al., 2008; Ito and Bartholow, 2009; Decety et al., 2010; Decety, 2011).

For example, Cheng and colleagues (2007) investigated the neural processes underlying expert and naïve populations' reactions to a person experiencing painful (penetrated with acupuncture needles) and non-painful (Q-tip) stimulation. Evidence from their fMRI investigation revealed increased activity for the pain matrix network (dACC, insula, somatosensory cortex) in naïve participants. On the other hand, the experts (physicians with acupuncture experience) provided no activity in these areas, instead neural activity was recorded in vmPFC which is involved in emotion regulation (Decety, 2011) and TPJ which has previously been implicated in self-other differentiation and ToM (Decety and Lamm, 2007). These results suggest that the acupuncturists could influence their vicarious pain experience by down-regulating these responses through emotional regulation and increased self-other differentiation. Using a similar paradigm, Decety et al. (2010) used EEG to identify the time course of empathic responses and the regulation thereof. The authors identified that for naïve participants, early (N110) and late (P3) activity showed differential responses for painful and non-painful stimuli but when the experienced physicians viewed this stimulus set, there were no differences in early or late processes which suggests that emotion regulation can impede on early processing of painful stimulus presentation (Decety et al., 2010).

Relevant to emotion regulation is the ability to inhibit explicit emotional reactions. It is important to regulate explicit emotional expressions to maintain egalitarian status within society. An example of this was shown in an fMRI study by Richeson and colleagues (2003) who argued that people (especially those with high racial bias) during interracial contact must inhibit racial attitudes and this would result in depletion of executive functions (i.e., response inhibition) which in turn would lead to impaired performance on a subsequent task that requires these functions. They tested this hypothesis by measuring White participants internal beliefs toward racial groups (Blacks and Whites) using an Implicit Association Test (IAT). Additionally, they asked participants to comment on a few questions with a Black Experimenter (mixed-race interaction) and then participants completed a Stroop task to measure executive functioning (task inhibition). Results showed that those who scored higher on the IAT for racial bias, also showed more interference effects on the subsequent Stroop task. When followed up with an fMRI task where participants were presented with Black and White faces, they found increased activation in the ACC and the dlPFC when Black faces were presented, suggesting greater response inhibition during these trials. A significant positive relationship was also found between the increase in ACC and dlPFC activation and the IAT and Stroop task, where this increase in the right dlPFC mediated the effect between IAT and Stroop interference. Collating this evidence, it suggests that people who show higher interracial bias try to inhibit automatic stereotypes, ultimately leading to a reduction in cognitive resources.

Another nice example of emotion regulation was shown in an fMRI study by Cunningham and colleagues (2004). They showed White participants pictures of Black (out-group) and White (in-group) faces either very briefly (30 ms) or for a longer duration (525 ms). The authors predicted that when these pictures would be presented very briefly, participants would not have enough time to regulate their emotions (i.e., negative responses to the Black faces). The fMRI results showed there was increased activation in the amygdala for Black faces compared to White faces when the stimuli were presented very briefly but no such effect was found when the stimuli were presented for longer. Instead they found increased activation in the dlPFC and ACC in the long stimulus presentation condition. When correlating the scores of an IAT regarding race bias with that of neural activity, a positive relationship was shown between behavioral data and fMRI activity in the amygdala for Black and White faces. Similarly, Black-White differences in amygdala activity between the short and long image presentations were predicted by frontal activation. Taking these findings together, it suggests that an automatic race bias against Black faces in White participants is moderated using reflective cognitive processes that only take effect after a period of time. Given that it is not socially acceptable to show explicit in-group bias, the authors interpreted this effect as increased emotion regulation of an automatic bias.

However, social categorization can also override automatic biases. For example, Van Bavel et al. (2008) investigated whether arbitrary and temporary novel group membership could override the effects of predominant group memberships within society (i.e., race as described in their study). Therefore, they randomly assigned participants to a mixed-race team. Pairing behavioral paradigms with functional MRI, the authors measured activity in the fusiform face area (FFA), which has previously been shown to be modulated by face perception and visual expertise (Gauthier et al., 1999, 2000; Golby et al., 2001; Van Bavel et al., 2011), when participants were presented with pictures of faces of in-group and out-group members. The results revealed greater activity in bilateral FFA for in-group faces compared to out-group faces. Interestingly this effect was specific to in-group vs. out-group and was not modulated by race (see also Van Bavel and Cunningham, 2009 and Van Bavel et al., 2011 for similar results). This provides evidence that categorizing people from a different race into an in-group can inhibit automatic racial biases.

## Conclusion

The current review aimed to highlight how group membership modulates the affective, cognitive, and regulative components of empathy. We have shown that in-group bias is not only a result of increased vicarious simulation of the actions (Gutsell and Inzlicht, 2010; Molenberghs et al., 2012b) and feelings (Xu et al., 2009) of in-group compared to out-group members but also follows from increased activation in ToM regions (Adams et al., 2009; Mathur et al., 2010; Cheon et al., 2011) when trying to understand the mental state of in-group vs. out-group members. These group biases can be influenced by emotional regulation (Ito and Bartholow, 2009) depending on expertise (Cheng et al., 2007; Decety et al., 2010) and context (Richeson and Shelton, 2003; Cunningham et al., 2004) so that we respond in a socially acceptable way to our environment. Lastly, it seems that arbitrary re-categorization can override automatic biases such as race (Van Bavel et al., 2008). Seeing as group membership modulates responses at each component of empathy, future investigations should identify methods of reversing these biases at each of the three distinguishable levels.

### Conflict of interest statement

The authors declare that the research was conducted in the absence of any commercial or financial relationships that could be construed as a potential conflict of interest.
